# Sicherheitsaspekte bei der Behandlung mit Clozapin: Unerwünschte Arzneimittelwirkungen, Titration und Therapeutisches Drug Monitoring – eine narrative Übersicht

**DOI:** 10.1007/s40211-023-00474-z

**Published:** 2023-06-22

**Authors:** Stefan J. Berger, Alex Hofer

**Affiliations:** Department für Psychiatrie, Psychotherapie, Psychosomatik und Medizinische Psychologie, Univ.-Klinik für Psychiatrie I, Medizinische Universität Innsbruck, Anichstr. 35, 6020 Innsbruck, Österreich

**Keywords:** Clozapin, Unerwünschte Arzneimittelwirkungen, Titration, Therapeutisches Drug Monitoring, Clozapine, Side Effects, Titration, Therapeutic Drug Monitoring

## Abstract

**Hintergrund:**

Laut geltender Leitlinien sollte Clozapin als Mittel dritter Wahl bei therapieresistenten schizophrenen Störungen (TRS) Verwendung finden. Im klinischen Alltag erfolgt der Einsatz jedoch häufig zu einem wesentlich späteren Zeitpunkt, was zu einer deutlichen Verschlechterung der Krankheitsprognose führt. Der erste Teil dieser narrativen Übersicht beleuchtet häufige unerwünschte Arzneimittelwirkungen (UAW) von Clozapin, die Bedeutung einer langsamen Titration und spezifische Aspekte des Therapeutischen Drug Monitoring (TDM).

**Material und Methoden:**

Die Datenbank Medline sowie die Guideline for the use of clozapine 2013 der Netherlands Clozapine Collaboration Group und die S3-Behandlungsleitlinie Schizophrenie der Deutschen Gesellschaft für Psychiatrie und Psychotherapie, Psychosomatik und Nervenheilkunde e.V. wurden nach relevanter Literatur untersucht, die letzte Abfrage erfolgte am 28.04.2023.

**Resultate:**

Trotz einzigartiger Wirksamkeit erfolgt die Verordnung von Clozapin im klinischen Alltag deutlich seltener als indiziert und variiert sowohl innerhalb als auch zwischen den Ländern. Neben hämatologischen, metabolischen und vegetativen UAW stellen die vor allem bei rascher Titration zu beobachtenden entzündlichen Erkrankungen in Form von Pneumonie oder Myokarditis wesentliche klinische Herausforderungen dar, so dass CRP-Kontrollen von besonderer Bedeutung sind. In diesem Zusammenhang muss insbesondere auch beachtet werden, dass Geschlecht, Rauchverhalten und ethnische Herkunft der Patient:innen den Clozapin-Stoffwechsel beeinflussen und daher eine individuelle Dosierung erforderlich machen.

**Schlussfolgerung:**

Eine nach Möglichkeit langsame Titration, TDM und gegebenenfalls eine CYP-Diagnostik erhöhen die Patient:innensicherheit während einer Behandlung mit Clozapin und somit die Wahrscheinlichkeit einer frühzeitigen Verordnung dieser Substanz bei TRS.

## Abstract (en)

**Background:**

According to current guidelines, clozapine should be used as a third step in treatment resistant schizophrenia (TRS). In everyday clinical practice, however, it is frequently used at a much later stage, which leads to a significant deterioration of prognosis. The first part of this narrative overview focuses on the most frequent side effects of clozapine, on the relevance of slow titration, and on specific aspects of therapeutic drug monitoring (TDM).

**Material and Methods:**

Medline, the Guideline for the use of clozapine 2013 of the Netherlands Clozapine Collaboration Group, and the S3 Guideline for Schizophrenia of the German Association for Psychiatry, Psychotherapy and Psychosomatics were searched for relevant literature, the last query dating from April 28th, 2023.

**Results:**

Despite its unique efficacy clozapine is underused in clinical practice and prescription varies between and within countries. Next to hematological, metabolic, and vegetative side effects, clozapine induced inflammation manifesting in the form of pneumonia or myocarditis, which is mainly associated with rapid titration, represents a major clinical challenge with CRP monitoring being of particular relevance. In this context, it also has to be noted that sex, smoking behavior, and ethnic origin impact clozapine metabolism, thus requiring personalized dosing.

**Conclusion:**

Slow titration when possible, TDM, and CYP diagnostics when appropriate increase patient safety during treatment with clozapine and thus the likelihood of early prescription of this compound in TRS.

## Einleitung

Clozapin gilt als Goldstandard in der Behandlung der therapieresistenten schizophrenen Störungen (TRS), d. h. bei unzureichendem Ansprechen auf zwei andere Antipsychotika in ausreichender Dauer (jeweils 6 Wochen) und Dosierung. Aufgrund potenziell schwerwiegender unerwünschter Arzneimittelwirkungen (UAW) verbietet sich sein Einsatz als Mittel erster oder zweiter Wahl, so dass im Vorfeld eine Pseudotherapieresistenz ausgeschlossen werden muss, die beispielsweise durch schlechte Therapieadhärenz, Maskieren der Response durch UAW oder Komorbiditäten bzw. durch die Psychose verstärkenden Substanzgebrauch bedingt sein kann [1].

Neben seiner herausragenden Wirkung in der Symptombehandlung der TRS besitzt Clozapin antiaggressive [2] und antisuizidale Eigenschaften [3] und kommt unter anderem auch bei Psychosen im Zuge der Lewy Body Demenz [4] oder des Morbus Parkinson [5] zur Anwendung. Des Weiteren wurde die Verordnung von Clozapin mit einem geringeren Hospitalisierungsrisiko [6, 7] und herabgesetzter Mortalität in Verbindung gebracht [8, 9], und dennoch kommt diese Substanz im klinischen Alltag häufig verzögert oder überhaupt nicht zum Einsatz [10, 11] Beispielsweise ergab eine kürzlich publizierte Studie, dass im Vereinigten Königreich lediglich ein Drittel der für eine Behandlung mit Clozapin geeigneten Personen diese tatsächlich erhält [12]. Die internationalen Verordnungszahlen reichen von 0,06 % in Japan bis 0,1–0,2 %/100 Patient:innen in Finnland, Deutschland oder Neuseeland [13], und auch die zum Einsatz kommende Dosierung variiert stark [14]. Die Gründe für diese zurückhaltende Verordnung von Clozapin sind vielfältig und beinhalten beispielsweise verschiedene Sorgen sowohl der Patient:innen als auch der behandelnden Ärzt:innen („Clozaphobie“) [15–17], wie etwa die Befürchtung von schweren UAW und die mit der Verordnung verbundenen unerlässlichen Monitoring-Untersuchungen (z. B. verpflichtende Bluttests), aber auch unzureichende Erfahrung [16, 18] und divergierende Titrationsschemata [19, 20]. Ziel der beiden Übersichtsarbeiten ist es, den Umgang und die Überwachung einer Behandlung mit Clozapin darzustellen und somit deren frühzeitigen Einsatz bei TRS zu unterstützen. Der vorliegende erste Teil stellt zusammenfassend häufige UAW dar, präsentiert einen Vorschlag für die Titration dieser Substanz im stationären und ambulanten Setting und gibt eine praktische Anleitung für die Durchführung des Therapeutischen Drug Monitoring (TDM) [21].

## Methodik

Erstmalig im März 2022 wurde in Medline die Suchformel (clozapine) AND (infection OR agranulocytosis OR thrombocytopenia OR cardiotoxicity OR cardiomyopathy OR myocarditis OR thromboembolism OR metabolic OR constipation OR seizures OR hypersalivation OR renal OR pharmacokinetics OR toxicity OR mortality OR titration) eingegeben. Da diese Abfrage über 7000 Einträge ergab, wurden die Suchbegriffe auf Titel eingeschränkt. Diese Suche wurde in weiterer Folge mehrmals wiederholt, zuletzt am 28.04.2023. Zusätzlich wurden spezifische Fragestellungen abgefragt und die Bibliografie der verwendeten Artikel nach weiteren Hinweisen durchsucht. Daneben wurden die im Internet veröffentlichte S3-Behandlungsleitlinie Schizophrenie der Deutschen Gesellschaft für Psychiatrie und Psychotherapie, Psychosomatik und Nervenheilkunde e.V. (DGPPN) und die Guideline for the use of clozapine 2013 der Netherlands Clozapine Collaboration Group hinzugezogen. Nach Screening auf Relevanz wurden 301 Arbeiten selektiert, auf deren Basis die vorliegende Übersicht erstellt wurde.

## Resultate

### Unerwünschte Arzneimittelwirkungen

#### Pneumonie

Auf Basis der Pharmakovigilanz-Datenbank der Weltgesundheitsorganisation berichtete eine rezente Übersichtsarbeit, dass von den dort erfassten Fällen, bei denen unter der Behandlung mit Clozapin eine Pneumonie auftrat, 30 % letal endeten [22]. Die Inzidenz der Clozapin-bedingten Pneumonien wurde unter anderem mit 21,4 pro 1000 Personenjahren angegeben bzw. eine Risikoerhöhung um 0,64 % bei Beginn der Behandlung mit Clozapin [23]. Die Pathophysiologie dieser UAW ist komplex. Neben einer Clozapin-bedingten Senkung des Immunglobulinspiegels und einer Up-Regulation des Interleukin-1-Rezeptors stellt das Krankheitsbild der TRS an sich in Kombination mit anderen Faktoren wie Rauchen, Adipositas und Adhärenzproblemen eine begünstigende Bedingung für das Auftreten einer Pneumonie dar. Zusätzlich erhöhen andere mit Clozapin verbundene UAW, wie Hypersalivation oder Sedierung, das Risiko für eine Aspirationspneumonie [24, 25]. Durch das Auftreten einer Pneumonie werden wiederum Zytokine freigesetzt, welche hemmend in den CYP-Metabolismus eingreifen und somit zu erhöhten Clozapin-Plasmaspiegeln beitragen können. Diese positive Rückkopplungsschleife führt zu einer sich aggravierenden Situation und begünstigt das Auftreten weiterer UAW [26–28].

#### Hämatologische UAW bis hin zur Agranulozytose

Eine Veränderung des weißen Blutbildes während einer Behandlung mit Clozapin ist keine Seltenheit. Bei einem Abfall der Leukozyten < 4000/mm^3^ liegt eine Leukopenie vor. Fällt die absolute Neutrophilenzahl < 1500/mm^3^, spricht man von einer Clozapin-induzierten Neutropenie (CIN), bei einem Wert < 500/mm^3^ von einer Clozapin-induzierten Agranulozytose (CIA). Die Inzidenz dieser Blutdyskrasien wurde mit 3,8 % (CIN) beziehungsweise 0,9 % (CIA) angegeben, die Letalität liegt bei 0,013 % [29]. Die Pathophysiologie ist auch in diesem Fall nicht eindeutig geklärt. Im Falle der Agranulozytose gibt es verschiedene Erklärungsversuche. Neben immunologischen Ansätzen, welche durch gesteigerte proliferative Reaktionen der mononukleären Zellen und durch das schnellere Auftreten bei Re-Exposition gestützt werden, werden eine direkte Toxizität von Clozapin gegen Stromazellen des Knochenmarks und eine Unreife der Neutrophilen diskutiert. Das multifaktorielle Zusammenspiel verdeutlicht die Notwendigkeit regelmäßiger Blutbild-Überwachungen [30, 31].

#### Kardiovaskuläre UAW

Die erhöhte kardiovaskuläre Mortalität unter Clozapin-Behandlung ist einerseits durch eine direkte kardiale Wirkung, andererseits durch den negativen Einfluss von Risikofaktoren bedingt [27].

##### Myokarditis

Auffallend bei dieser UAW sind die erheblichen geografischen Inzidenz-Unterschiede. Während die Inzidenz in der australischen Population beispielsweise auf etwa 2 % geschätzt wird [32], liegt diese bei Nicht-Australier:innen bei etwa 0,3 % [33]. Dies ist wohl zu einem großen Teil auf eine gesteigerte Bewusstheit und entsprechende Überwachung zurückzuführen, welche zu einer im Vergleich zu anderen Regionen häufigeren Diagnose einer Myokarditis in der australischen Population führt. Ein großes Problem der Clozapin-bedingten Myokarditis stellt die mannigfaltige Präsentation dar. Nur durch ein zielgerichtetes Monitoring ist ein Erkennen der häufig unspezifischen Symptome, wie z. B. Fieber, Müdigkeit oder Dyspnoe, möglich [34]. Die Sterblichkeitsrate bei adäquatem Monitoring wird mit < 10 % angegeben [32]. Die Pathogenese dieser UAW ist bis dato nicht eindeutig geklärt, als Risikofaktoren gelten neben höherem Alter und gleichzeitiger Einnahme von Natriumvalproat eine zu rasche Auftitration und/oder Poor Metabolizer-Status, der vor allem bei Patient:innen mit asiatischer Abstammung bzw. der amerikanischen Urbevölkerung zu finden ist [35]. Dadurch kann es zur Freisetzung von Zytokinen kommen, die wiederum das an der Metabolisierung von Clozapin hauptsächlich beteiligte CYP1A2 hemmen und dadurch zu erhöhten Clozapin-Plasmaspiegeln führen können. Wird die Titration fortgesetzt, kann es zur Bildung von Autoantikörpern kommen, die letztlich eine Myokarditis oder eine andere entzündliche Erkrankung auslösen [36].

##### Kardiomyopathie

Auch im Fall dieser UAW sind signifikante Inzidenz-Unterschiede auffallend. Diese werden zwischen 0 und 12 % angegeben und sind ebenfalls durch unterschiedliche Diagnosepfade bedingt. Meist handelt es sich um eine dilatative Kardiomyopathie, häufigstes Symptom ist eine belastungsabhängige Dyspnoe mit oder ohne Tachykardie. Diagnose-erschwerend ist, dass das C‑reaktive Protein (CRP) und die Troponin-Werte häufig unauffällig sind und andere Herzenzyme eine zweifelhafte Rolle spielen. Ist ein ursächlicher Zusammenhang mit der antipsychotischen Behandlung gesichert, muss Clozapin abgesetzt werden. Es wird angenommen, dass unter adäquater Medikation gute Chancen auf eine Besserung der linksventrikulären Funktion bestehen [32]. Die Pathogenese ist wiederum ungeklärt, vermutet wird, dass neben einer direkten Clozapin-bedingten Toxizität persistierende Tachykardie, veränderte Herzfrequenzvariabilität und erniedrigte Selenspiegel eine Rolle spielen. Als Risikofaktoren gelten Adipositas, Diabetes mellitus, Alkoholmissbrauch und viele weitere [37].

##### Orthostatische Dysregulation und Tachykardie

Eine Behandlung mit Clozapin führt bei 24 % der Patient:innen zu einer Hypotension [32] und bei 54 % zu einer kompensatorischen Tachykardie [38]. Bedingt wird dies durch die ausgeprägte Blockade von α1-Rezeptoren, aggravierend wirken sich eine rasche Titration und eine hohe Dosis aus. Höheres Alter, kardiovaskuläre Vorerkrankungen und eine antihypertensive Vorbehandlung zählen zu den bekannten Risikofaktoren. Eine Behandlungsoption stellt die Gabe von Betablockern dar [38, 39].

#### Metabolische UAW

Es ist bekannt, dass die Lebenserwartung von Menschen mit schizophrenen Störungen bis zu 20 Jahre verkürzt ist. Dies ist besonders auf chronische Herzerkrankungen und metabolische Veränderungen, wie z. B. Diabetes mellitus, zurückzuführen [40].

##### Gewichtszunahme

Es gibt unzählige Angaben zur Inzidenz und Prävalenz einer Clozapin-bedingten Gewichtszunahme. Fakt ist, dass unter dieser Behandlung ein besonders hohes diesbezügliches Risiko besteht und in der überwiegenden Mehrzahl der Studien die Häufigkeit mit deutlich > 60 % angegeben wird [41, 42].

Pathophysiologisch spielt neben der Affinität von Clozapin zu mehreren Neurotransmittersystemen eine genetische Komponente eine wesentliche Rolle. Hauptverantwortlich für die Gewichtszunahme unter Clozapin-Einnahme ist der Antagonismus am Histaminrezeptor (H1), der mit einer gesteigerten Nahrungsaufnahme einhergeht [43].

##### Hyperlipidämie, pathologische Glucosetoleranz und Diabetes mellitus

Unter der Behandlung mit Clozapin wurden sowohl Veränderungen im Glukose-Stoffwechsel mit einem erhöhten Risiko für prädiabetische und diabetische Zustände als auch ein gehäuftes Auftreten einer Hypertriglyceridämie nachgewiesen. Diese metabolischen Entgleisungen führen im Extremfall zu einem metabolischen Syndrom [32]. Die Wahrscheinlichkeit für das Auftreten eines metabolischen Syndroms ist bei Männern unter Clozapin-Behandlung um den Faktor 1,38, bei Frauen um den Faktor 2,51 erhöht [44]. Es existieren verschiedene Theorien zur Pathogenese, wie z. B. eine direkte Toxizität auf Beta-Zellen im Pankreas oder die direkte Beeinträchtigung des Insulinsignalweges in Muskelzellen. Vor allem bei Patient:innen mit einer bereits vor Clozapin-Behandlungsbeginn eingeschränkten Beta-Zell-Aktivität führt dies zu Problemen im Glukose-Stoffwechsel [32].

#### Vegetative UAW

Diese sind hauptsächlich durch die starke anticholinerge und α -adrenolytische Wirkung von Clozapin bedingt und reichen von Hypersalivation bis zu intestinalen Problemen [45].

##### Hypersalivation

Während einer Behandlung mit Clozapin berichten 48 % der Patient:innen über eine untertags auftretende Hypersalivation und 85 % über entsprechende nächtliche Beschwerden [46]. Pathophysiologisch wird neben einer agonistischen Wirkung von Clozapin am muskarinischen M_4_-Rezeptor auch eine Blockade vagaler Rezeptoren diskutiert. Diese kann zur Beeinflussung des vagal gesteuerten unwillkürlichen Schluckvorgangs führen [47].

##### Obstipation

Bei 25 % der Patient:innen tritt vor allem bei körperlicher Inaktivität, ballaststoffarmer Ernährung und geringen Trinkmengen eine Clozapin-induzierte gastrointestinale Hypomobilität (CIGH) auf [48, 49]. Pathophysiologisch spielt neben der anticholinergen auch die antiserotonerge Wirkung von Clozapin eine Rolle.

#### Zentralnervöse Störungen

##### Schläfrigkeit – Sedierung – Schwindel

Der sedierende Effekt von Clozapin stellt eine der häufigsten UAW dar, tritt bei 49,6 % der Patient:innen auf und ist meist von kurzer Dauer [50, 51]. Pathophysiologisch ist die antihistaminerge Wirkung von Clozapin dafür verantwortlich. Eine hohe Dosis und morgendliche Verabreichung gelten als Risikofaktoren [52].

#### EEG und Krampfanfälle

EEG-Veränderungen treten bei mehr als der Hälfte der mit Clozapin behandelten Patient:innen auf [21]. Die Häufigkeit von Krampfanfällen (meist tonisch-klonisch) ist abhängig von der Dosis, bei 300 mg/d wird diese mit 1 % angegeben, bei > 600 mg/d mit 4,4 % [53].

Eine Senkung der Anfallsschwelle tritt bei fast allen Antipsychotika auf. Bei Patient:innen mit bekannter Epilepsie oder zerebraler Vorschädigung ist die Indikation für den Start einer Behandlung mit Clozapin deshalb genau abzuwägen [54].

#### Fieber und malignes neuroleptisches Syndrom (MNS)

##### Fieber

Zu Beginn einer Clozapin-Behandlung tritt bei ca. 5 % der Fälle eine vorübergehende Erhöhung der Körpertemperatur ein. Die betroffenen Patient:innen sollten vor allem hämatologisch untersucht werden, um Komplikationen frühzeitig zu erkennen. Die Temperaturerhöhung an sich ist jedoch eine harmlose und passager auftretende UAW [55].

##### MNS

Bei sehr hohem Fieber sollte ein MNS ausgeschlossen werden. Dieses tritt bei 0,2 % der Patient:innen unter Clozapin-Behandlung auf [56]. Pathophysiologisch könnte die Blockade von D_2_-Rezeptoren im Hypothalamus und im Striatum dafür ursächlich sein [57].

### Titration

Wie im Vorfeld bereits erwähnt wurde, beträgt die Ereignisrate für das Auftreten einer Clozapin-induzierten Myokarditis in Australien etwa 2 %, während in 15 anderen Ländern eine Ereignisrate von 0,3 % beobachtet wurde [58, 59]. Dieser Unterschied wird u. a. auf die deutlich langsamere Titration in europäischen Ländern zurückgeführt.

Neben der Myokarditis stellen auch das Auftreten einer Pneumonie, Serositis, Pneumonitis, Hepatitis, Pankreatitis, Nephritis oder Kolitis besondere Gefahren bei Verwendung eines aggressiven Titrationsschemas dar [56]. UAW, wie Hypersalivation, Sedierung und Schluckstörung, von denen jede für sich das Risiko einer Aspirationspneumonie erhöht, sind ebenfalls mit einer schnellen Titration verbunden [60]. Beispielhaft zeigt Tab. 1 das in der niederländischen Leitlinie empfohlene Clozapin-Titrationsschema im stationären und ambulanten Setting [61]. Zu beachten ist, dass die Zieldosis für Patient:innen mit asiatischer Abstammung ungefähr halb so hoch ist wie bei Kaukasier:innen (siehe „Interpretation des TDM“) [33, 62].

**Table 1:** 

Woche 1		Woche 2	
*Stationäres Setting*			
Tag 1	2 × 12,5 mg	Tag 8	150 mg
Tag 2	50 mg	Tag 9	150 mg
Tag 3	75 mg	Tag 10	150 mg
Tag 4	100 mg	Tag 11	200 mg
Tag 5	100 mg	Tag 12	200 mg
Tag 6	100 mg	Tag 13	200 mg
Tag 7	100 mg	Tag 14	200 mg
*Ambulantes Setting*			
Tag 1	2 × 12,5 mg	Tag 8	75 mg
Tag 2	25 mg	Tag 9	100 mg
Tag 3	25 mg	Tag 10	100 mg
Tag 4	50 mg	Tag 11	100 mg
Tag 5	50 mg	Tag 12	100 mg
Tag 6	50 mg	Tag 13	100 mg
Tag 7	75 mg	Tag 14	100 mg

Die ersten Zeichen einer Clozapin-bedingten Hypersensitivitätsreaktion sind CRP-Erhöhung und Fieber. Vor Behandlungsbeginn und in den ersten 4 Behandlungswochen bzw. während der Titration sollte deshalb auf jeden Fall eine CRP- und Leukozyten-Überwachung stattfinden. CRP ist kostengünstig, ubiquitär verfügbar und nach aktuellem Wissensstand das erste Zeichen einer Clozapin-induzierten Entzündung [55]. Poor Metabolizer, welche bereits während der ersten Behandlungswoche gefährdet sind, können bei CRP-Erhöhung frühzeitig erkannt werden und mit einer angepassten, besonders vorsichtigen Titration vor dem Auftreten gravierender UAW bewahrt werden [33, 63].

### Therapeutisches Drug Monitoring (TDM)

Grundsätzlich ermöglicht das TDM, eine optimale und möglichst geringe Erhaltungsdosis zu finden, unter der die Patient:innen ausreichend therapeutische Wirksamkeit erfahren, gleichzeitig aber ein möglichst geringes Risiko für das Auftreten von UAW haben. Zusätzlich kann die Therapieadhärenz überwacht werden [64].

Die Steady-State Talspiegel von Clozapin sollten zwischen 350 und 600 ng/ml liegen [65, 66], die gleichzeitige Einnahme von potenten Inhibitoren (z. B. Fluvoxamin) oder Induktoren (z. B. Carbamazepin) sollte vermieden werden [67]. Im Fall von Fluvoxamin kann eine Dosisreduktion auf 1/5 bis auf ein 1/10 der Normaldosis notwendig sein [68, 69], was allerdings durchaus auch therapeutisch genutzt werden kann.

#### Durchführung

Das TDM wird grundsätzlich unter Standardbedingungen durchgeführt, wofür Steady-State-Talspiegel-Bedingungen benötigt werden. Der Steady-State wird nach 5 Halbwertszeiten erreicht. Im Falle von Clozapin, das eine Halbwertszeit von ungefähr 12 h besitzt, liegt ein Steady-State-Talspiegel somit nach rund 3 Behandlungstagen vor. Bei einmaliger täglicher Einnahme liegt der Talspiegel 10–14 h nach der letzten Einnahme [70], bei zweimaliger täglicher Einnahme vor Einnahme der morgendlichen Dosis [71]. Bei der Planung der Durchführung des TDM ist zu beachten, dass die Plasmaspiegel in den ersten Behandlungstagen oft keiner linearen Kinetik folgen. Zudem werden Störfaktoren, wie Entzündungen, Rauchverhalten, Kaffeekonsum oder induktiv beziehungsweise inhibitorisch wirkende Substanzen, nicht berücksichtigt [27].

#### Interpretation des TDM

Treten bei Standarddosierung im Zuge des TDM erhöhte Serum‑/Plasmakonzentrationen auf, ist dies ein Hinweis auf einen erniedrigten Clozapin-Stoffwechsel. Ursächlich dafür können mehrere Faktoren, wie z. B. die Einnahme von CYP1A2-Inhibitoren, Nichtraucher:innen-Status, Entzündungen, Adipositas oder weibliches Geschlecht sein [54]. Erniedrigte Serum‑/Plamakonzentrationen treten hingegen bei erhöhtem Clozapin-Stoffwechsel auf, wie z. B. bei Einnahme von CYP1A2-Induktoren, Raucher:innen-Status, männlichem Geschlecht, europäischer oder westasiatischer Abstammung [72].

Patient:innen mit asiatischer Abstammung benötigen eine Clozapin-Mindestdosis von 175 mg/Tag (weibliche Nichtraucherinnen) bis 300 mg/Tag (männliche Raucher) [73, 74], jene mit europäischer Abstammung hingegen Tagesdosen zwischen 265 mg/Tag (weibliche Nichtraucherinnen) und 525 mg/Tag (männliche Raucher) [33, 75].

## Conclusio

Bei gesicherter TRS ist und bleibt Clozapin Antipsychotikum der Wahl und zeichnet sich im Vergleich zu anderen oralen Antipsychotika durch eine besondere Wirksamkeit gegen Positiv- und Negativsymptome [76], antisuizidale [3] und antiaggressive Eigenschaften [2] sowie durch ein geringeres Hospitalisierungsrisiko aus [6, 7]. Daneben war die Behandlung mit Clozapin in einer landesweit an mehr als 60.000 Patient:innen durchgeführten Studie aus Finnland mit einem Beobachtungszeitraum von bis zu 20 Jahren sowohl im Vergleich zur Nicht-Behandlung als auch zur Behandlung mit anderen Antipsychotika mit der geringsten Mortalität verbunden [9].

Trotz der genannten Vorteile erfolgt die Verordnung von Clozapin im klinischen Alltag deutlich seltener als indiziert [12, 77] und variiert sowohl innerhalb als auch zwischen den Ländern [78–80]. Beispielsweise ergab eine in verschiedenen europäischen Ländern durchgeführte Untersuchung, dass in Ungarn lediglich 11 % der TRS-Patient:innen mit Clozapin behandelt wurden, in Polen, Estland und der Slowakei bis zu 35 %, in Kroatien 41 % und in Slowenien und Serbien mehr als 60 % [81]. Die Gründe für diese zögerliche Verordnung wurde sowohl in mangelnder Erfahrung im Umgang mit Clozapin [16, 18] als auch in verschiedenen Bedenken von Seiten der Patient:innen und der behandelnden Ärzt:innen verortet [2–4]. Um dem entgegen zu wirken, wurden in der vorliegenden Arbeit zunächst die häufigsten UAW von Clozapin basierend auf ausgewählter Fachliteratur zusammengefasst.

Auf Grund der verpflichtenden Monitoring-Untersuchungen werden Clozapin-bedingte Veränderungen des weißen Blutbildes (CIN, CIA) heutzutage in der Regel rasch erkannt und sind gut beherrschbar, und auch bei metabolischen UAW stehen verschiedene pharmakologische und nicht-pharmakologische Behandlungsmöglichkeiten zur Verfügung [82]. In den vergangenen Jahren wurde jedoch vermehrt auf das Risiko von entzündlichen Erkrankungen (Pneumonie, Myokarditis) hingewiesen, denen vermutlich die Freisetzung von Zytokinen mit nachfolgender Hemmung des Clozapin-Metabolismus und in weiterer Folge eine Erhöhung des Plasmaspiegels zugrunde liegen [36]. Dieser Mechanismus wurde insbesondere bei rascher Titration beobachtet, so dass in dieser Arbeit exemplarisch das von der niederländischen Leitlinie entnommene langsame Titrationsschema dargestellt wurde [61], zumal eine mit einer Clozapin-Intoxikation vergesellschaftete Pneumonie mit deutlich erhöhter Letalität verbunden ist [22]. Entsprechend sollte vor Beginn und in den ersten 4 Behandlungswochen bzw. während der Titration neben der Leukozyten-Überwachung eine CRP-Kontrolle stattfinden. Zu beachten ist in diesem Zusammenhang auch, dass Geschlecht, Rauchverhalten und ethnische Herkunft der Patient:innen den Clozapin-Stoffwechsel beeinflussen, so dass individuelle Dosierungen erforderlich sind [33] und dem TDM und gegebenenfalls einer CYP-Diagnostik eine besondere Bedeutung zukommt.
